# Circulating Tumor DNA in the Management of Early-Stage Breast Cancer

**DOI:** 10.3390/cells12121573

**Published:** 2023-06-07

**Authors:** Katerina Vlataki, Sevastiani Antonouli, Christina Kalyvioti, Evangeli Lampri, Sevasti Kamina, Davide Mauri, Haralampos V. Harissis, Angeliki Magklara

**Affiliations:** 1 Department of Clinical Chemistry, Faculty of Medicine, University of Ioannina, 45110 Ioannina, Greece; kathy_dv@yahoo.com (K.V.); arella_935@hotmail.com (S.A.); 2 Breast Unit, Department of Surgery, University Hospital of Ioannina, 45500 Ioannina, Greece; christinakalivioti@gmail.com (C.K.); hharisis@uoi.gr (H.V.H.); 3 Department of Pathology, University Hospital of Ioannina, 45500 Ioannina, Greece; elampri@uoi.gr (E.L.); sevastikamina@yahoo.com (S.K.); 4 Department of Medical Oncology, University Hospital of Ioannina, 45500 Ioannina, Greece; dmauri@uoi.gr; 5 Biomedical Research Institute, Foundation for Research and Technology, 45110 Ioannina, Greece; 6 Institute of Biosciences, University Research Center of Ioannina (URCI), 45110 Ioannina, Greece

**Keywords:** liquid biopsies, circulating tumor DNA, circulating free DNA, early-stage breast cancer, digital PCR, next-generation sequencing, prognosis, therapy monitoring, minimal residual disease, patient surveillance

## Abstract

Liquid biopsies refer to the isolation and analysis of tumor-derived biological material from body fluids, most commonly blood, in order to provide clinically valuable information for the management of cancer patients. Their non-invasive nature allows to overcome the limitations of tissue biopsy and complement the latter in guiding therapeutic decision-making. In the past years, several studies have demonstrated that circulating tumor DNA (ctDNA) detection can be used in the clinical setting to improve patient prognosis and monitor therapy response, especially in metastatic cancers. With the advent of significant technological advances in assay development, ctDNA can now be accurately and reliably identified in early-stage cancers despite its low levels in the bloodstream. In this review, we discuss the most important studies that highlight the potential clinical utility of ctDNA in early-stage breast cancer focusing on early diagnosis, detection of minimal residual disease and prediction of metastatic relapse. We also offer a concise description of the most sensitive techniques that are deemed appropriate for ctDNA detection in early-stage cancer and we examine their advantages and disadvantages, as they have been employed in various studies. Finally, we discuss future perspectives on how ctDNA could be better integrated into the everyday oncology practice.

## 1. Introduction

The steady increase in breast cancer (BrCa) incidence since the mid-2000s [1] has become a major issue in oncology, as it translates to more than 2 million new cases annually around the globe [2], a number expected to reach 3 million by 2040 [3]. Notably, the mortality rate has decreased in most Western countries due to earlier diagnosis through mammography screening and increased awareness, accompanied by significant advances in treatment [4]. Nonetheless, BrCa remains the primary cause of cancer-related death in the female population worldwide, with over 0.6 million women dying of the disease in 2020 [2]; this number is predicted to rise to ~1 million in 2040 [3]. Recent statistics from USA show that at the time of diagnosis, the vast majority of women have local- (64%) or regional- (27%) stage BrCa, while only 6% present with distant metastasis [5]. In the same population, the 5-year relative survival rate is 99% for women diagnosed with localized cancer, and it drops down to 30% for those with de novo metastatic disease [6]. However, approximately 30% of the patients who respond well to locoregional treatments will relapse [7], with a significant proportion of them developing incurable metastases.

To date, the standard practice for BrCa detection in the asymptomatic population is based on routine mammography, usually starting at 50 years of age, often supplemented by an ultra-sound, even though there is no consensus yet on the use of the latter [8]. However, one should bear in mind that these imaging techniques may produce false-positive or false-negative results, leading to unnecessary biopsies in the first case or to under-diagnosis in the second [8]. Other shortcomings of this approach include the lower age limit for mammography screening and the low sensitivity and specificity of the technique. Any suspicious mammographic findings must be further investigated by tissue biopsy, which is considered the gold standard in diagnosis and provides important information with prognostic and predictive value that guides therapeutic strategies. Nevertheless, tissue biopsy has inherent limitations since it is an invasive, time-consuming procedure, rendering its frequent use prohibitive, especially in patients with compromised health. Consequently, therapeutic decisions are commonly made based on the molecular testing of a single biopsy of the primary tumor or of one metastatic lesion, disregarding the way in which cancer cells evolve and change their genetic makeup over time. The fact that only a handful of tumor regions are sampled during biopsy hampers its utility even more since it fails to capture spatial intra-tumor heterogeneity, which may lead to incomplete tumor profiling and misinformed therapeutic planning. 

Current treatment approaches for early-stage BrCa consist of a complex combination of local modalities (surgery, radiotherapy) with a pharmacological scheme (chemotherapy, endocrine and/or targeted therapy) directed at preventing recurrence and metastatic disease [8]. There are currently no validated biomarkers to monitor patient response to therapy in real time, therefore escalation or de-escalation strategies cannot be employed during the course of treatment to improve therapeutic outcomes. After completion of therapy, the standard of care for patient follow-up involves periodic check-ups that include physical examination and routine mammography or other imaging tests aiming to detect early tumor relapse. The limitations of this protocol were underlined by the recent ESMO guidelines, where it was stated that “*very importantly*, *most available data for follow-up recommendations come from an era of less sophisticated diagnostic procedures*… *and new trials are urgently needed to reassess this question*” [8]. 

In conclusion, there is an unmet clinical need for novel, personalized biomarkers that will allow for a more efficient management of early-stage breast cancer, from screening to follow-up, materializing the promise of precision medicine and leading to improved patient outcomes. Liquid biopsies have emerged as a source of such biomarkers in oncology and have rapidly evolved in the last 10 years fueled by remarkable technological advances in genome analysis.

**Figure 1:**
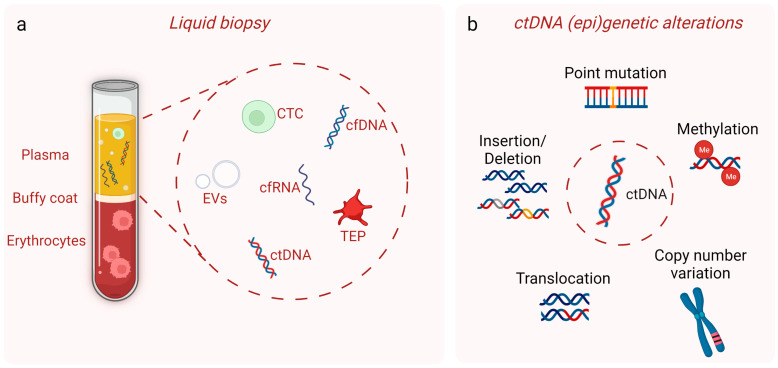
(**a**) Blood is the most commonly used body fluid for liquid biopsies. The most studied liquid biopsy biomarkers are depicted, namely circulating tumor cells (CTCs), circulating tumor DNA (ctDNA), cell-free RNA (cfRNA), extracellular vesicles (EVs) and tumor-educated platelets (TEPs). Cell-free DNA (cfDNA) is also shown. (**b**) ctDNA carries the same genomic (point mutations, insertions/deletions, translocations, copy number variations) and epigenomic (DNA methylation) alterations as the tissue tumor DNA. This image was created with the use of BioRender (https://biorender.com/) (accessed on 20 April 2023).

Liquid biopsies refer to the isolation and analysis of tumor-derived biological material from body fluids, most commonly blood, in order to provide clinically valuable information for the management of cancer patients [9]. Even though the term first referred to circulating tumor cells, it now encompasses circulating tumor DNA (ctDNA), circulating cell-free RNA and extracellular vesicles released from cancer cells, as well as tumor-educated platelets (Figure 1a) [10,11]. The most appealing trait of liquid biopsies is their non-invasive nature that allows for serial sampling without raising patient safety concerns. The analysis of circulating tumor components, especially of ctDNA, originating from multiple tumor regions provides a molecular footprint of the whole tumor entity and enables real-time monitoring of tumor evolution. For further reading on liquid biopsies in general as well as their advantages and disadvantages compared to conventional tissue biopsy, the reader is referred to some excellent reviews in the field [12,13,14].

In the past years, several studies have demonstrated that ctDNA detection can be introduced to the clinical setting to improve patient prognosis and monitor therapy response, especially in advanced and metastatic cancers [15,16,17,18]. Currently, ctDNA testing is used in clinical practice for genotyping advanced cancers to identify actionable mutations and guide therapy, particularly in cases where tumor tissue is not available [19]. Studies performed in early-stage cancers were lacking in number, and only in recent years sensitive enough assays have been developed for reliable ctDNA detection in these patients.

The main goal of this review is to provide a comprehensive summary of the most significant studies on ctDNA analysis in patients with early-stage BrCa, focusing on its potential utility in diagnosis, therapy monitoring, detection of minimal residual disease (MRD) and metastatic relapse. We highlight the strengths and weaknesses of these studies and discuss future perspectives and challenges for implementing ctDNA testing in this setting. A big part of ctDNA analysis is the methodological approach used and its potential to provide precise and reliable data; for this reason, special emphasis is put on the technical parameters of each study.

## 2. Circulating Tumor DNA

The presence of cell-free nucleic acids in the bloodstream was described for the first time in diseased and healthy individuals in 1948 [20]. Thirty years later, by employing a sensitive radioimmunoassay, it was demonstrated that cancer patients had elevated serum levels of cell-free DNA (cfDNA), suggesting that this might be a new valuable biomarker for their prognostic and therapeutic evaluation [21]. It was later confirmed, in 1989, that tumor-derived circulating DNA was a sub-fraction of the cfDNA in cancer patients [22].

Circulating cfDNA is released during cell death processes, such as apoptosis or necrosis, but it may also be actively secreted by cells in extracellular vesicles (reviewed in [23]). In healthy individuals, more than 90% of cfDNA originates from the hematopoietic cells [24]. In cancer patients, the fraction of cfDNA that is composed of tumor-derived DNA varies depending on tumor burden [25], and it ranges from ≤0.01–0.1% in early-stage to ≥5–10% in advanced-stage cancers [26]. It should be noted, though, that the amount of ctDNA shedding into the bloodstream depends not only on the size of the tumor but also on its location and vascularity as well as on the presence of metastases, leading to variable ctDNA levels even across patients at the same disease stage [26]. Tumor DNA, both in tissue and in circulation, is defined by the presence of genetic and epigenetic alterations such as somatic point mutations, copy number variations (CNVs), chromosomal rearrangements or specific methylation patterns (Figure 1b) (reviewed in [27]). Consequently, analysis of ctDNA, isolated in a non-invasive manner, can be used for tumor molecular profiling, providing the same information as the genomic DNA extracted from cancer cells obtained by tissue biopsy [26]. Another distinctive feature of cfDNA from healthy cells compared to ctDNA is their fragmentation pattern. Even though both DNA species are highly fragmented, cfDNA shows a median length of 167 bp, while ctDNA is 20–30 bp shorter, with the etiology underlying this size difference still being unclear (reviewed in [28]). Interestingly, accumulating evidence suggests that cfDNA size profiling, so as to separate the fraction enriched for ctDNA, can improve the sensitivity of ctDNA analysis techniques (reviewed in [28]). The short half-life time of ctDNA in the bloodstream, ranging from 16 min to 2.5 h [29], offers the opportunity for a real-time assessment of the current disease state. 

The first study convincingly demonstrating the clinical utility of ctDNA was published in 2014 [30]; ctDNA analysis in metastatic colorectal cancer patients showed extremely high sensitivity and specificity for the detection of *BRAF* and *KRAS* mutations [30]. Following this, numerous clinical studies were performed investigating the biomarker value of ctDNA in various types of cancer yielding encouraging results (reviewed in [9,10,27,31]).

At present, the wider clinical application of ctDNA is mainly hampered by its low levels in the bloodstream, requiring highly sensitive techniques for precise and reliable identification and quantification [26]. This becomes an even greater challenge in the early stages of cancer, when the ctDNA is found in minute amounts in biological fluids due to the small tumor burden [25]. However, recent technological advances in assay development (see Section 3) have achieved extremely low limits of detection, providing the biomedical community with the necessary means to proceed to the next step, the evaluation of the clinical utility of ctDNA in early-stage cancers in large clinical trials.

## 3. Techniques Used for ctDNA Detection and Analysis in Early-Stage Cancers

**Figure 2:**
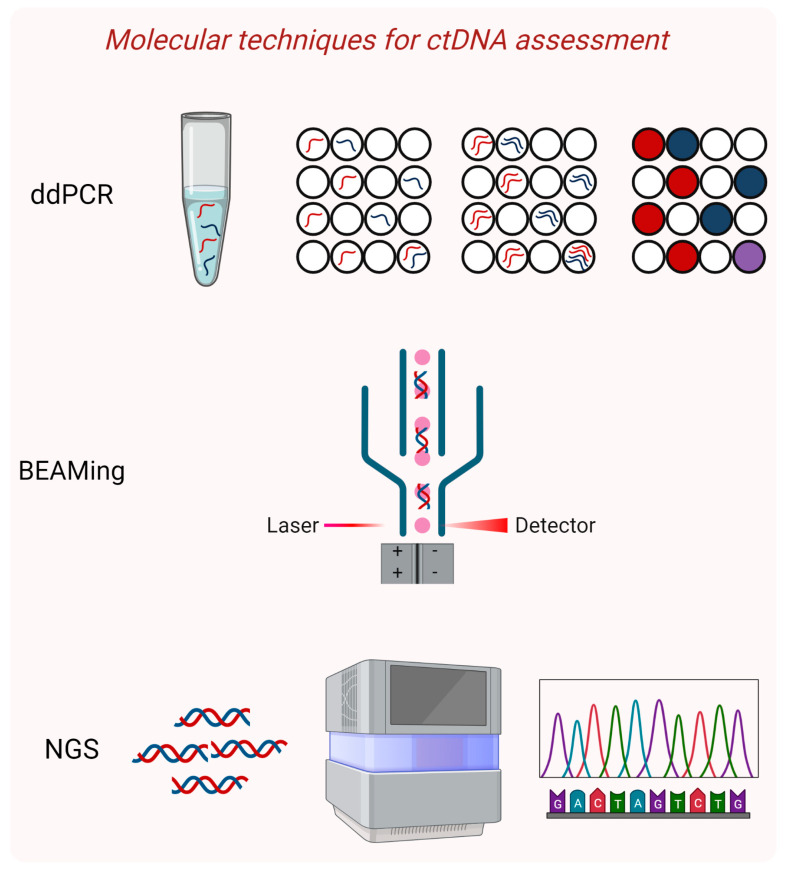
Highly sensitive molecular techniques for ctDNA detection and characterization. Droplet digital PCR (ddPCR) is based on the partitioning of the sample into millions of water-in-oil droplets, so that each droplet contains a single DNA molecule that is amplified individually. BEAMing stands for beads, emulsion, amplification, and magnetics and is a type of dPCR, where amplified wild-type and mutant alleles are differentially labeled and are separated through flow cytometry. Next-generation sequencing (NGS) approaches that employ massive parallel sequencing with improved technical and bioinformatics protocols to reduce errors have gained ground in ctDNA analysis. This image was created with the use of BioRender (https://biorender.com/) (accessed on 20 April 2023).

The ultra-sensitive techniques that have been effectively applied for the identification of ctDNA in the early stages of tumor development include digital PCR and modified NGS-based methodologies (Figure 2) that can reliably detect rare alleles down to 0.01% [26].

Digital PCR (dPCR) is an exceptionally powerful technique that allows for accurate quantification of rare mutations as it was first demonstrated by Kinzler and Vogelstein who coined the term [32]. Its basic principle is the over partitioning of a sample into a multitude of units, so that each unit contains one DNA molecule that is amplified by an individual PCR. In this way, the exponential, analog signal of the traditional PCR is transformed into a linear, digital one, which is transmitted by each amplified DNA molecule and is easily detected [32]. Two main dPCR platforms have been implemented successfully in liquid biopsies, BEAMIng and droplet dPCR. 

BEAMing stands for beads, emulsion, amplification and magnets and, as its name indicates, it combines emulsion PCR and flow cytometry of magnetic beads. After a targeted amplification step to enrich for the desired sequences, individual DNA molecules are attached to magnetic beads and further amplification occurs in numerous water-in-oil emulsion droplets [33]. At the end, each droplet contains one bead coated with thousands of fluorescently labeled copies of the same DNA molecule. Since mutant and wild-type alleles carry different fluorophores, they can be separated and quantified through flow cytometry. This method can identify low-abundance variants with confidence, even when present at a frequency of less than 0.01% [34]. BEAMing has been applied successfully in oncological patients, including ones with breast cancer, mainly in the metastatic or advanced setting [35,36,37]. It should be noted that the BEAMing assay has been commercialized and it can only be performed by trained personnel in accredited centers. Based on our experience [38], it is a rather costly technique that involves a complicated and quite laborious protocol, which is probably hard to implement in everyday clinical practice.

Droplet digital PCR (ddPCR) also relies on the partitioning of the sample into millions of water-in-oil droplets so that each droplet contains a single DNA molecule that is amplified individually. Mutant and wild-type alleles are differentially labeled with fluorescent TaqMan-based probes and identified by flow cytometry [39]. This system was successfully used for accurate measurement of germline copy number variations, detection of rare alleles at a 0.001% frequency and absolute quantification of plasma cfDNA in a cost-effective and practical way [39]; soon after, it was widely applied in clinical research.

Several initial studies in patients with early-stage breast cancer served as a proof of concept for the feasibility of dPCR assays to detect ctDNA in this context, with some of them focusing on the analysis of mutations in *PIK3CA* [40,41,42], a gene frequently found altered in the primary stages of the disease. *PIK3CA* mutations were detected at similar percentages (~25%) in the ctDNA of early-stage TNBC [40] and primary BrCa patients [41] before undergoing surgery. Three hotspot mutations were examined by two groups, with the first one using the QX200 Droplet Digital PCR System [40] and the second one applying a microfluidics-based dPCR platform (QuantStudioTM3D) [41]. A much higher sensitivity (93%) and a 100% specificity in detecting these mutations was achieved by a different group when they employed a ddPCR assay (RainDance Technologies Inc., Lexington, MA, USA) in pre-surgical plasma samples of early-stage BrCa patients [42]. Notably, even after surgery, 50% (5 out of 10) of the mutation-positive patients examined still had detectable ctDNA, indicating incomplete tumor elimination [42]. The noticeable difference in sensitivities in the aforementioned studies is probably due to the different dPCR platforms used; the RainDance Tehnologies system is reported to partition the sample into 1 billion individual units affording higher sensitivity levels compared to other dPCR platforms (reviewed in [43]). In later years, the use of ddPCR was widely adopted for the analysis of ctDNA isolated from patients with early-stage BrCa during disease monitoring, providing substantial evidence for the clinical utility of the new biomarker. These studies are discussed in greater detail in Section 4.1.

The unparallel sensitivity of dPCR has earned it the title of the “gold standard” in ctDNA analysis; however, its utility is limited by the small number of genomic loci that can be examined in one run. This pushed the research community to the development of highly sensitive NGS techniques that could reach comparable detection limits with dPCR but could also track numerous mutations at the same time, reducing the possibility of false-negative results.

Apart from BEAMing, Kinzler’s and Vogelstein’s group pioneered the Safe-Sequencing System (Safe-SeqS), which is based on the introduction of a unique identifier (UID) to each DNA molecule to be analyzed by NGS [44]. After amplification, many daughter molecules with the same UID are generated, which should all carry the original mutation, if that was present in the template molecule. In this way, reads that carry PCR or sequencing errors are identified and excluded from further analysis. The authors demonstrated that this approach could be used to identify rare variants and it could be integrated into any sequencing pipeline [44]. Employment of this technique in plasma samples of stage II colon cancer patients after tumor resection identified a group with detectable ctDNA that had a very high risk of recurrence [45]. In the group of ctDNA-positive patients who did not receive adjuvant chemotherapy, the sensitivity and specificity of ctDNA in predicting tumor relapse at 36 months were 48 and 100%, respectively. The authors reported plasma variants with allele frequencies (AFs) down to 0.003% underlining the power of this technique [46].

Cancer personalized profiling by deep sequencing (CAPP-Seq) is another ultrasensitive NGS method for identification and quantification of ctDNA present in low levels [47]. It relies on an improved library preparation protocol for low DNA input and on an innovative bioinformatics approach to design “selectors”, which are biotinylated DNA oligonucleotides that target frequently mutated regions in the type of cancer under investigation. The technique is first applied to tumor tissue to identify mutations in each patient and then directly to plasma samples to detect and quantify ctDNA. As a proof of principle, the authors used CAPP-Seq in non-small cell lung cancer patients, with a sensitivity of 50% in stage I and 100% in stage II–IV patients, and a specificity of 96% in both groups [47]. The limit of detection of the assay was 0.02% showing its potential to be used in early-stage cancers. Another advantage of this method is the lack of the need to use an individualized assay for each patient since CAPP-Seq is designed to cover mutations for almost all patients with a specific cancer type. In this way, this approach significantly saves time and reduces the overall cost.

Forshew and colleagues developed tagged-amplicon deep sequencing (TAm-Seq), a method that is based on the generation of amplicons that tile large genomic regions of cancer-associated genes in short segments [48]. This is performed in two steps: an initial preamplification step using a pool of target-specific primers is followed by selective amplification in individual PCRs. As a proof of concept, the authors designed primers to amplify large coding regions of *TP53*, *PTEN*, *EGFR*, *BRAF*, *KRAS*, and *PIK3CA*, and by applying Tam-Seq they were able to identify plasma mutations at 2% AF with 97% sensitivity and specificity in patients with high-grade serous ovarian carcinomas [48]. More recently, an improved platform that used enhanced TAm-Seq (eTam-Seq) was described [49]. The original assay had been expanded to cover more cancer-associated genes based on a primer design scheme that allowed more efficient amplification of highly fragmented DNA, such as ctDNA. The bioinformatics pipeline was also upgraded for better base calling and improved detection of single nucleotide variants (SNVs), short insertions/deletions (indels) and CNVs. Analytical validation of the assay demonstrated high sensitivity and specificity for detection of mutations at 0.25% AF and excellent concordance with digital PCR, confirming that it can be used for reliable identification of rare variants [49]. The Tam-Seq technology has now been commercialized by Inivata (Research Triangle Park, NC, USA), which offers the RaDaR (Residual Disease and Recurrence) assay based on the InVision^®^ liquid biopsy platform.

A technique called targeted error correction sequencing (TEC-Seq) was developed by Velculescu’s group based on targeted capture of multiple genomic regions and deep sequencing [50]. The authors took several steps to optimize their technical protocol as well as their bioinformatics analysis so that they could remove potential errors and reliably detect rare alleles. They applied this method in plasma samples from 194 untreated patients with early or advanced breast, colorectal, lung or ovarian cancer to analyze mutations in 58 genes. The assay showed a 100% specificity and was able to detect mutant alleles down to 0.1% AF. However, only 62% of patients with localized disease (stages I and II) were positive for ctDNA, a rather low sensitivity that could be improved by deeper sequencing, better error correction methods, higher DNA input and serial testing, as the authors noted [50].

Overall, both dPCR and NGS-based assays have set high expectations for their successful application in the management of early-stage cancer patients. DPCR techniques are ultrasensitive and can consistently detect rare tumor-associated variants. Their main pitfall is that they require a priori knowledge of the interrogated genomic loci and can only assay a few mutations at a time. CAPP-seq, Tam-Seq and TEC-seq rely on deep sequencing of a specific panel of common driver genes that can be applied in most tumor types and their performance is comparable to that of dPCR. The use of unique molecular identifiers (UMIs) to reduce PCR amplification errors can be incorporated in any sequencing protocol and has proven to be a valuable tool for the detection of ultra-low frequency mutations with high accuracy.

## 4. Circulating Tumor DNA in the Management of Early-Stage Breast Cancer

**Figure 3:**
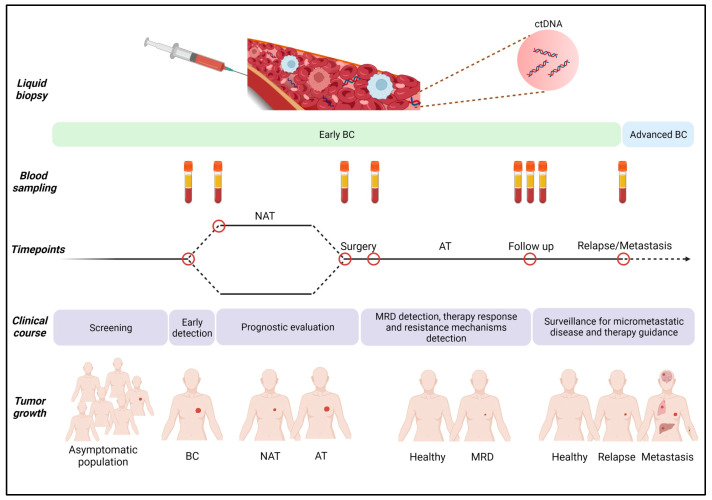
Analysis of ctDNA at various time points can contribute to the clinical management of early-stage breast cancer (BC). Early diagnosis by ctDNA detection could be followed by administration of neoadjuvant therapy (NAT), and ctDNA testing in post-surgical samples could help in the detection of minimal residual disease (MRD). Longitudinal ctDNA analysis in patients after NAT and/or adjuvant therapy (AT) could also be used in monitoring therapy response, early detection of resistance and prediction of metastatic relapse. This image was created with the use of BioRender (https://biorender.com/) (accessed on 22 April 2023).

Repetitive blood sampling at specific time points during the course of cancer allows the usage of ctDNA in diagnosis, prognosis and recurrence risk prediction; when ctDNA is analyzed at regular time intervals, it can provide real-time monitoring of disease progression and patient response to therapies (Figure 3). In this section, we discuss the findings of the most important studies where ctDNA has been examined in patients with early-stage breast cancer.

### 4.1. ctDNA in Early BrCa Diagnosis

Undoubtedly, the most desired but also most challenging application of ctDNA is its potential use in the early diagnosis of BrCa, since its obtention via non-invasive methods makes it an appealing marker for screening the asymptomatic population. In this direction, a few pilot studies have offered preliminary but promising results [51,52]. Rodriguez et al. employed Safe-Seq to detect mutations in patients with suspicious mammography findings before they underwent tissue biopsy [51]. Specifically, tumor profiling of 29 patients revealed *PIK3CA* mutations in 79.3% (23/29) and *TP53* in 34.5% (10/29) of them. One third of the patients (10/29) also carried plasma mutations, mostly at very low AFs (0.05%–3.60%), as expected at this early stage of the disease. Higher plasma ctDNA levels tended to be present in patients with more aggressive clinicopathological characteristics. Interestingly, out of the 13 plasma mutations detected in total, 9 were concordant with tissue mutations and 4 (3 in *TP53* and 1 in *PIK3CA*) were found only in plasma [51]. These three *TP53* alterations have been annotated as driver mutations in the COSMIC database and were missed by tissue biopsy. Despite the small number of patients included, and the fact that only two genes were examined, the results of this study corroborated the potential utility of integrating plasma ctDNA detection in the screening process for breast cancer and underlined its power to capture intratumoral heterogeneity in contrast to tissue biopsies.

A cohort of 152 patients with suspicious mammographic or ultrasound findings were included in a different prospective study to investigate the potential of ctDNA as a clinically actionable biomarker [52]. Fifty of them were ultimately diagnosed with benign disease and the rest (*n* = 102) with early-stage BrCa and in need of chemotherapy. In the cancer patients, plasma samples were collected before surgery, 2 days and 3 weeks after surgery and at the end of chemotherapy. Two different breast-cancer-associated gene panels were designed, with the first one covering 341 hotspots in 68 genes and the second one covering the entire coding regions of 136 genes. The smaller panel was used to sequence the tissue and plasma samples from 36 patients, and almost all of them (35/36) had at least one somatic mutation, while ctDNA mutations were identified in 19 of them (52.8%). Of the 66 patients tested using the larger gene panel, all had at least one tissue mutation, and 49 of them (74.2%) presented with ctDNA mutations in their pre-surgical plasma samples. When the authors integrated the ctDNA results with the corresponding imaging scores for predicting the presence of cancer, they estimated a positive predictive value (PPV) of 92.45% (49/53), a sensitivity of 74.24% (49/66) and a specificity of 92% (46/50). These results suggested that ctDNA testing could complement imaging techniques to improve early breast tumor diagnosis and avoid unnecessary biopsies in patients with suspicious findings. Τhe authors also noted that the detection power of ctDNA for early BrCa diagnosis increased with the number of interrogated genetic loci in plasma samples and the depth of sequencing. It should be mentioned that a relatively large percent of plasma mutations (33.5%) was not detected in tissue, and several mutations were suspected to be the product of clonal hematopoiesis of indeterminate potential (CHIP), a suspicion that was confirmed only in selected cases [52]. CHIP refers to somatic mutations arising in clones of blood cells with no other findings for hematologic neoplasias, and it has been strongly associated with age [53]. CHIP mutations may present a serious impediment in ctDNA analysis leading to false-positive results [53]. This can be avoided by sequencing a matching buffy coat sample when ctDNA testing is performed, a step that should be considered in all relevant clinical studies. 

The above-described work demonstrated the feasibility of applying ctDNA analysis in early BrCa diagnosis. However, an indispensable tool to accomplish such an ambitious aim is the development and standardization of highly sensitive assays capable of finding a needle in a haystack given the very low frequency of cancer-related mutant alleles in the blood of patients at the initial stages of the disease [26]. Furthermore, long-term, multi-center clinical trials are needed to establish the clinical utility of ctDNA in early BrCa detection and determine whether it should be integrated into national screening programs.

### 4.2. ctDNA in Prognosis and Prediction of Therapy Response in the Neoadjuvant Setting

At present, many early-stage BrCa patients are offered neoadjuvant therapy (NAT), which includes pre-operative systemic treatment aiming to reduce the extent of resection and increase the likelihood of breast-conserving surgery by downstaging tumors [54]. However, only 30% of the patients treated achieve complete pathological response (pCR), which is associated with improved patient disease-free and overall survival [55]. There are currently no approved biomarkers for monitoring response during treatment [54], even though early identification of good and poor responders could allow for timely, personalized, therapeutic interventions, sparing the patients who would not benefit from NAT from the unnecessary side-effects. The following studies offer substantial evidence that ctDNA detection in the neoadjuvant setting could be associated with treatment response.

Riva et al. focused on early-stage TNBC patients receiving NAT and examined the clinical utility of ctDNA analysis [56]. After identifying *TP53* tissue mutations by NGS, the authors designed customized ddPCR assays to track these mutations in plasma samples collected at various time points during patient monitoring. At baseline, ctDNA was detected in 75% (27/36) of patients and it dropped during treatment in all of them but one, in whom it was increased. Notably, this patient was the only one whose cancer progressed during chemotherapy, underlining the potential clinical value of ctDNA as a marker of therapy response and tumor progression. The authors did not find a significant correlation between clinical response, pCR and ctDNA detection at any time point. After surgery, ctDNA was no longer detectable in any patients [56], even though some of them presented metastatic relapses during follow-up. The fact that the authors monitored only one mutation per patient might explain these findings, as it is now well established that multiple plasma mutations should be followed to avoid false negative results.

This was indeed shown in the work of Cavallone and colleagues who adopted a similar approach, following 26 TNBC patients but monitoring on average five mutations per patient [57]. They designed and applied customized dPCR assays for detection of plasma mutations in samples obtained before, during and after neoadjuvant chemotherapy (NAC), based on each patient’s tumor tissue profiling [57]. At baseline, ctDNA was detected in 96% of patients, showing that interrogation of several genomic loci could significantly improve assay sensitivity (compared to 75% in previous study). Plasma ctDNA levels before NAC were strongly associated with aggressive tumor features [57]. At the beginning and after completion of treatment, ctDNA was detectable in ~70% of patients, but it was significantly associated with the presence of residual disease at the time of surgery only in the first time point. The absence of detectable ctDNA in the pre-surgical samples was associated with long-term relapse-free and overall survival. Specifically, patients who tested positive for ctDNA at that time point had a median time to relapse or to death 21 and 35 months, respectively, and this was not reached by patients with undetectable ctDNA (median follow-up time: 63 months). These results confirmed that ctDNA testing of early-stage TNBC patients receiving NAC might have prognostic and predictive value, as long as 4–5 variants are monitored to ensure high sensitivity of detection [57].

The utility of ctDNA in monitoring NAT response and predicting outcome was also examined in patients with HER2^+^ early-stage BrCa who were treated with targeted therapy in the context of the NeoALTTO clinical trial [58]. The study included 69 patients, and the presence of ctDNA was assessed in plasma samples received before NAT, after 2 weeks of treatment and prior to surgery. The authors used ddPCR to monitor only one mutation (in the *PIK3CA* or *TP53* gene) in each patient based on their tumor tissue results. ctDNA was detected in 41% (28/69), 20% (13/65) and 5% (3/60) of patients at the three time points, respectively. The presence of plasma mutation at baseline was significantly associated with decreased probability of achieving pCR. Another interesting finding was that the patients with HER2-enriched subtype, as determined by PAM50 testing, who had undetectable ctDNA at baseline had the highest pCR rates compared to the other subtypes, suggesting that these patients may be good candidates for de-escalation therapies [58]. Furthermore, it was shown that patients who were still ctDNA-positive after 2 weeks of NAT had a lower pCR rate. The authors failed to detect any significant associations between ctDNA detection and event-free survival, which might have been due to the fact that these analyses were underpowered. It should be mentioned that a main limitation of this study was the monitoring of only one mutation in plasma samples; investigation of more mutations might have increased the number of ctDNA-positive patients. Even so, the results delivered by this study strongly support a role for ctDNA detection in monitoring patient response to anti-HER2 treatment as well as in patient risk stratification, possibly enabling personalized therapy with improved outcomes [58].

In a cohort of 84 early-stage BRCa patients of all major subtypes who were treated with NAC but were at high risk of metastatic relapse based on MammaPrint testing, ctDNA detection was examined as a biomarker of therapy response [59]. The patients had enrolled in the multicenter neoadjuvant I-SPY 2 TRIAL, and ctDNA analysis was performed retrospectively in plasma samples collected before (T0), during (T1 and T2) and after the end of NAC treatment (T3). For ctDNA detection, the authors used personalized panels interrogating 12–16 highly ranked somatic mutations that had been previously identified through whole-exome sequencing (WES) of tumor tissue samples [59]. Notably, the sequencing of matching buffy coat samples was performed to exclude germline and CHIP variants from analysis. By applying ultra-deep NGS (>90,000×) and using a cut-off of two detected plasma variants to call a sample ctDNA-positive, the researchers determined that 73% of the patients had detectable ctDNA before treatment initiation. The ratio of ctDNA-positive patients was higher in the TNBC and HER2^+^ subtypes and ctDNA was also associated with tumor size [59]. On average, ctDNA positivity decreased during therapy down to 9% after completion of NAC, and the authors proceeded to evaluate ctDNA clearance as a predictor of response to NAC. Out of the 56 ctDNA-positive patients at T0, 29 (52%) had detectable ctDNA at T1 and 83% of them (24/29) did not obtain pCR, which was a significantly higher number compared to the ones who cleared ctDNA at T1 (52%, 14/27) and still had residual disease at surgery. Notably, the PPV of ctDNA positivity in predicting failure to achieve pCR increased with time, suggesting that ctDNA analysis after completion of NAC may be used for patient risk stratification and for planning adjuvant treatments [59].

Finally, recent work investigated the utility of ctDNA to predict therapy response in patients who received tecemotide, a synthetic lipopeptide that is used in antigen-specific cancer immunotherapy added to neoadjuvant treatments [60]. Tissue testing by a 93-gene panel informed the design of individualized assays in 145 patients. Plasma samples were collected before, during and at the end of NAT and they were assayed using SiMSen-seq (Simple, Multiplexed, PCR-based barcoding of DNA for SENsitive mutation detection using sequencing). This is a high-resolution sequencing approach that allows detection of variant alleles at a < 0.1% frequency and can be tailored to a patient’s molecular profile [61]. For most patients, 1–2 mutations were tracked in their plasma samples, and only in 31 patients (21.4%) 3–6 mutations were analyzed. ctDNA was present in 43% of patients (63/145) at baseline, in ~40% of them at mid-therapy (25/63) and in ~24% (15/63) at the end of NAT. Notably, 96.8% (30/31) of patients that were ctDNA-positive during therapy were non-responders, suggesting that ctDNA could be used as a negative predictor of response to NAT. Detection of ctDNA at mid-therapy was also associated with the lack of pCR [60]. It is noteworthy that a recent meta-analysis that included most of the studies discussed above [56,57,58,59] concluded that ctDNA detection in early-stage BrCa patients receiving NAT was significantly associated to worse relapse-free and overall survival both at baseline and after the end of treatment [62]. However, the authors did not detect a significant association between ctDNA detection and pCR achievement. This meta-analysis largely confirms the data described in this section that support a role for ctDNA analysis in patient stratification during NAT [62].

### 4.3. ctDNA in MRD Detection and Patient Surveillance

Adjuvant therapy refers to the post-operative treatment administered to cancer patients and is the backbone of the therapeutic regimens in early-stage breast cancer. It is meant to eliminate MRD that could lead to tumor recurrence and advanced metastases that are practically incurable [63]. Detection and monitoring of MRD through the employment of a reliable biomarker would be critical for assessing the success of surgical resection and for identifying patients at risk who would truly benefit from adjuvant therapy [63]. Since there is no such an approved marker, it is currently recommended to administer systemic treatments after surgery to most early-stage BrCa patients [8], even though this practice improves the outcome only in a small percentage of them and often leads to severe side effects [64]. 

Over the last years, several studies analyzing plasma mutations in early-stage BrCa patients receiving adjuvant and/or neoadjuvant treatments have highlighted the potential of ctDNA in detecting MRD and predicting metastatic recurrence in these clinical settings. The patient cohorts examined in the following studies were of mixed or only hormone-receptor (HR)-positive BrCa subtypes (Table 1).

In one of the earlier studies in 20 patients who had received adjuvant therapy, post-surgical tracking of ctDNA identified metastatic recurrence with high sensitivity (93%) and specificity (100%) [65]. For the purpose of this study, the authors performed a low-pass whole-genome sequencing of tumor samples to identify chromosomal rearrangements and then proceeded to design highly sensitive personalized ddPCR assays so that they could analyze 4–6 of them per patient in serial plasma samples. ctDNA was detected in 13 out of the 14 patients who ended up developing metastases, and in 12 of them, the average lead time of molecular relapse was 11 months before clinical manifestations. Notably, none of the six patients with long-term, disease-free survival ever presented with detectable ctDNA during the course of the study [65]. 

Along the same lines, a prospective observational study sought to investigate the utility of tracking ctDNA as a biomarker for predicting the risk of metastatic relapse in early-stage breast cancer patients receiving NAT [66]. In a cohort of 55 patients, tumor biopsies were subjected to NGS for 14 breast cancer driver genes leading to the identification of at least one somatic mutation in 78% of them. Subsequently, ddPCR personalized assays designed to detect the mutations found in tissue were used to track ctDNA in serial plasma samples taken at baseline, 2–4 weeks after surgery and every 6 months during follow-up. ctDNA was identified in 69% of baseline samples and its levels correlated with histological grade and ER^−^ status, but its detection was not predictive of disease-free survival. In the samples collected shortly after surgery, ctDNA was detected only in 19% of the patients (7/37), and it was predictive of early relapse. This predictive value was enhanced when ctDNA was assessed in all post-operative samples for each patient, increasing its sensitivity and resulting in its detection at 7.9 months (median lead time) before clinical relapse. Notably, 80% (12/15) of patients who relapsed were identified as ctDNA-positive during mutation-tracking versus 50% who were found ctDNA-positive when only the first postoperative sample was considered. It is worth pointing out that the three patients who had undetectable ctDNA but relapsed presented only brain metastases. It has long been established that plasma ctDNA detection is challenging in patients with brain cancer, and cerebrospinal fluid sampling may be a better approach to characterize tumor genomic alterations [25]. Despite the small number of patients and the relatively short follow-up time (2 years), the conclusions reached in this study were significant as they clearly showed that tracking ctDNA mutations could effectively detect MRD and identify patients at high risk for tumor recurrence. In a small number of patients (*n* = 4), the authors also showed by deep targeted sequencing of plasma samples before relapse that they had new mutations different than the ones detected in the primary tumor and similar to the ones found in the metastatic lesions [66]. Since some of these mutations were potentially targetable, these results could potentially be used for personalized therapeutic interventions before patients reach an incurable advanced metastatic stage of disease.

The same group expanded their findings in a later study that recruited a larger number of early-stage BrCa patients receiving adjuvant or neoadjuvant therapy (*n* = 101) [67]. They used the same methodology as before, namely targeted NGS to uncover tissue mutations followed by personalized dPCR assays designed to track individual somatic mutations in plasma samples. Analysis of blood samples obtained at diagnosis and before initiation of treatment detected ctDNA in 51.2% (41/80) of patients at low variant allele frequencies (median VAF 0.36%) and was associated with a risk of relapse. The TNBC patients had the highest ctDNA levels at diagnosis, followed by the HER2^+^, while the ER^+^ patients had the lowest levels. During follow-up (median 35.5 months), ctDNA was detected in 16 patients (median VAF 0.16%) with a median lead time of 10.7 months before clinical relapse. Notably, detection of ctDNA during follow-up was highly prognostic in all major breast cancer subtypes. To enhance the power of their analysis, the authors combined the results of this study with the ones from their previous work [66]. In total, 88.4% (23/29) of patients who relapsed had detectable ctDNA, suggesting that molecular relapse detection is a clinically valid approach to identify patients at high risk for metastatic recurrence [67].

A more recent study provided further support to the clinical utility of ctDNA to predict metastatic relapse in early-stage BrCa patients who had undergone surgery and had received adjuvant therapy [68]. In this case, the authors relied exclusively on sequencing techniques to identify and monitor plasma mutations. Initially, they performed exome profiling of tumor tissues to identify all relevant mutations and then applied ultra-deep personalized targeted plasma sequencing for 16 of them. This approach enabled them to detect ctDNA in 89% (16/18) of the patients who relapsed. Molecular relapse preceded clinical relapse up to 2 years with a median of 8.9 months. None of the 31 non-relapsed patients were ctDNA-positive at any time during the course of the study (100% specificity) [68]. This work demonstrated convincingly that ultra-deep sequencing using a customized gene panel allows for a very low limit of detection (<0.01%), which is equivalent to the one reached by dPCR, and may yield similar—if not better—results regarding sensitivity and specificity. In accordance with the previous studies, the authors concluded that their findings advocated in favor of using ctDNA detection as a predictor of metastatic recurrence, which could possibly mandate an early therapeutic intervention with a second-line treatment.

A study discussed previously [59] also examined the utility of ctDNA in predicting metastatic relapse and survival in the neoadjuvant setting. The authors showed that patients who were ctDNA-positive during and after NAC had a significantly increased risk of metastatic recurrence compared to those who were ctDNA-negative. All patients (*n* = 17) who achieved pCR (100%) were ctDNA-negative and showed favorable distant disease-free survival (DRFS). Out of the 43 patients who did not achieve pCR, the ones who were still ctDNA-positive (*n* = 6) after completion of treatment had significantly worse DRFS than those who had cleared ctDNA (*n* = 37). This finding suggests that the ctDNA status of these patients after NAC could be used for risk stratification and for planning therapeutic strategies in the adjuvant setting. When a multivariable Cox regression analysis was performed taking into account pCR and breast cancer subtypes, detectable ctDNA after NAC was found to be a significant prognostic factor of poor DRFS [59].

The most prevalent breast cancer subtype is the hormone receptor-positive (HR^+^) subtype, which is highly treatable in its early stages; however, the risk of disease relapse remains steady from 5 to more than 20 years after diagnosis [75] As a matter of fact, most of the HR^+^ patients who present distant metastases do so after the first 5 years as a result of undetected residual disease [76]. Recently, the results of a prospective study designed to assess the association of ctDNA with metastatic recurrence in the late adjuvant setting in HR^+^ BrCa were reported [69]. Tumor tissue profiling through WES for 83 high-risk stage II-III HR^+^ BrCa patients who were diagnosed at least 5 years before enrolling in the study informed the design of personalized RaDaR assays (see Section 3) to track plasma mutations (median of 36) in order to detect MRD. Blood samples were obtained at baseline (time of enrollment) and during routine follow-up visits every 6–12 months (median follow-up time from baseline was 2 years). Four patients (5%) were found ctDNA-positive at baseline, and eight (10%) had detectable ctDNA at any time point with a median VAF of 0.0425%. Six patients (7.2%) developed distant metastases and they all were ctDNA-positive, with the median lead time of molecular relapse before clinical recurrence being 12.4 months. Recurrence-free survival for patients who had detectable ctDNA was worse compared with that of patients who were ctDNA-negative. The sensitivity of ctDNA to predict any clinical recurrence was 85.7%, with a specificity of 97.4%, a negative predictive value (NPV) of 98.7% and a PPV of 75%. When only distant metastases were considered, the sensitivity and NPV were both 100%. Limitations of this study include the infrequent sampling, the low number of recurrences probably due to the short follow-up and the lack of imaging results that cannot exclude the possibility that some patients might have had occult metastatic disease at the time of entry. Still, this was the first investigation that showed a strong association between molecular MRD, as indicated by ctDNA-positivity, with distant metastatic relapse more than 5 years after initial diagnosis of high-risk HR^+^ BrCa [69].

The issue of assay sensitivity to monitor molecular MRD was addressed by Parsons and colleagues who developed a highly sensitive and specific sequencing test based on the simultaneous tracking of hundreds of patient-specific mutations [70]. Analytical validation of their approach showed that it was 100 times more sensitive than ddPCR when tracking 488 mutations, and this was further validated in BrCa patients recently diagnosed with a metastatic disease (clinical sensitivity 81%). However, when their approach was applied in a cohort of 142 patients with early-stage BrCa, clinical sensitivity dropped to 23% (7/30) and 19% (6/32) when the plasma samples received shortly or approximately one year after surgery respectively were tested. The authors explained this drop in sensitivity as a consequence of the small number of tumor mutations they identified in tissue and tracked in ctDNA in most patients (median of 53 mutations) [70]. Overall, the assay had a median lead time (the time from a ctDNA-positive test to metastatic recurrence) of 18.9 months, which was a significant improvement compared to previous studies [67,68], but this might have been due to the much longer follow-up (median 7.1 years). Even though this study had several limitations, such as few plasma samples per patient, plasma processing was not specifically performed for ctDNA analysis, etc., it did provide some useful insights into proper assay development for MRD testing. The authors performed a thorough analytical validation of their technique and determined its analytical sensitivity and specificity [70]; these are the parameters often lacking in the studies, and especially the knowledge of the limit of detection of an assay is essential for the interpretation of a negative result. Finally, the authors clearly demonstrated the superiority of tracking many personalized mutations in ctDNA [70] so as to increase assay sensitivity, an approach that seems most promising in monitoring MRD in early-stage BrCa patients.

The same issue of assay sensitivity to detect residual disease in women who completed NAT was also addressed by McDonald et al. who developed a targeted digital sequencing (TARDIS) approach for the analysis of patient-specific mutations [71]. In order to achieve high sensitivity, TARDIS employs deep sequencing of multiple mutations, improved library preparation methods with the use of UMIs that reduce technical errors (see Section 3), and an advanced bioinformatics analysis pipeline. Evaluation of the analytical performance of the assay using reference samples showed 91% and 53% sensitivity at 0.03% and 0.003% VAFs, respectively, with 96% specificity. Importantly, the TARDIS results were extensively verified by dPCR [71]. For clinical validation of the assay, the authors first identified somatic mutations by WES in tissue biopsies from 33 women with stage I–III breast cancer, 22 of whom received NAT. Subsequently, the researchers applied TARDIS to monitor 6–115 mutations per patient in blood samples obtained before, during and after NAT completion. ctDNA was detected in all patients (32/32) before treatment initiation (0.11% median AF). After completion of treatment, ctDNA was detected in 77.3% of patients (17/22), with 92% of them (12/13) presenting with residual disease and 55.5% (5/9) reaching complete pathological response (pCR), as indicated by the absence of cancer cells in the resected tissue. ctDNA levels were lower in patients who achieved pCR (median AF 0.003%) compared to patients with residual disease (median AF 0.017%) [71]. 

Even though the above studies included small patient cohorts, they unequivocally showed that longitudinal analysis of multiple mutations in the cfDNA of early-stage BrCa patients is a valid approach to detect MRD and predict metastatic relapse. An important conclusion reached in these studies was that an increased number of plasma samples analyzed and of mutations monitored per patient are prerequisites to achieve the high assay sensitivity required in this setting in order to generate reliable data. These findings need to be tested now in large clinical trials that will evaluate whether planning therapeutic strategies based on ctDNA detection, and before the development of incurable metastases, can lead to improvement of patient outcome.

### 4.4. ctDNA in the Prediction of Relapse in TNBC Patients with Residual Disease

TNBC patients are more likely to present with metastatic relapse and die compared to those with the other BrCa subtypes [77]. These patients are commonly treated with NAT, and the presence of residual disease after completion of therapy has been associated with worse survival rates [78]. Studies have shown that ctDNA may have prognostic value in such patients and it could be used to select the ones who would mostly benefit from the available adjuvant therapies [72,73,74] (Table 1).

The potential of ctDNA to predict relapse in TNBC patients with residual disease after receiving NAC was examined in two studies published by Radovich and colleagues [72,73]. In both cases, the populations under study had enrolled in completed clinical trials. In the first study, blood samples were collected from 38 patients while they were receiving adjuvant therapy [72]. Tissue genomic analysis using a targeted NGS panel that interrogated 134 genes revealed mutations in 87% of the patients (33/38). NGS analysis of plasma samples detected ctDNA only in 4 of the 13 patients who relapsed (31% sensitivity, 100% specificity). Even though the authors noted that disease burden and the volume of plasma used were important factors that determined sensitivity [72], it should be pointed out that the NGS assay they used per se might not have been sensitive enough to detect ctDNA in this setting. This assumption is based on the coverage reported in paper [72], which was probably not sufficient to achieve the low limit of detection necessary for the reliable identification of molecular residual disease. 

A few years later, a preplanned secondary analysis of plasma mutations was performed in 142 early-stage TNBC patients [73], who had previously participated in a large multi-center prospective randomized trial. Blood samples were collected before administration of adjuvant therapy, and ctDNA sequencing was performed using one of two different Foundation Medicine targeted gene panels that had been thoroughly validated for ctDNA testing [79]. A total of 63.4% (90/142) of patients had detectable ctDNA; this was significantly associated with an inferior distant disease-free survival, disease-free survival and overall survival compared to the ctDNA-negative patients (median clinical follow-up 17.2 months) [73]. Collectively, the results of these studies [72,73] indicated that ctDNA could be used as a marker for molecular MRD as well as for patient stratification in the post-neoadjuvant setting. Future trials should aim to show whether administration of targeted therapy to TNBC patients, based on their ctDNA findings after surgery, could improve therapeutic outcomes.

The results of one such prospective study designed to assess the clinical utility of ctDNA in early-stage TNBC patients were just published (c-TRAK TN trial, NCT03145961) [74]. This multicenter phase II trial had two main objectives: (a) to assess whether employment of ctDNA assays could identify patients with molecular residual disease and (b) to evaluate the effect of pembrolizumab in ctDNA-positive patients. It enrolled 208 patients with early-stage TNBC and residual disease following NAC and surgery, or stage II/III given adjuvant chemotherapy. Personalized dPCR assays were designed for 161 of them based on one or two mutations identified by tumor tissue sequencing. CHIP variants were excluded by assaying a matching buffy coat sample. These patients entered ctDNA surveillance with blood samples collected at baseline (up to 12 weeks after NAT and surgery or adjuvant chemotherapy) and every 3 months for 2 years. A total of 27.3% of patients had ctDNA detected during the first 12 months. When a patient was ctDNA-positive, they were randomly allocated to an observation or an intervention group, and the patients in the latter group were administered pembrolizumab given that no metastatic recurrence was evident. Of the nine patients eligible to receive pembrolizumab, only five agreed to do so; none of them achieved ctDNA clearance at 6 months, and all subsequently relapsed with a median lead time between ctDNA detection and disease recurrence of 1.6 months. In the observation group, ctDNA clearance after 6 months occurred in 21.4% of patients (3/14), and the median lead time was 4.1 months. An interesting observation in this study was that many patients had already developed metastatic disease at the time of ctDNA detection, and the authors estimated that several of them had already undetected metastases when they enrolled. Thus, the authors urged for the application of more sensitive imaging techniques and for an earlier testing of ctDNA in future studies. Other improvements in the design of similar clinical trials that could be beneficial in detecting ctDNA before metastatic relapse would include shorter times between sampling and an increase in assay sensitivity by tracking more mutations [74].

## 5. Conclusions-Future Perspectives

The above studies clearly show that substantial support has been generated for the use of ctDNA as a clinically applicable biomarker in the early stages of breast cancer. Its utility in MRD detection, patient stratification, therapy guidance and response is now being evaluated in more than a dozen clinical trials (Table 2). In what is probably the most ambitious of these trials, STRIVE, ctDNA will be evaluated as a screening tool for early detection of breast and other cancers in thousands of asympromatic women undergoing mammography (Table 2). As the results of this study are awaited, other work has shown that a multi-analyte approach to early cancer diagnosis may be a more efficient way to tackle this issue. A test called CancerSEEK that combines detection of eight circulating protein markers with the interrogation of 1933 genomic loci in plasma was applied in 1005 patients diagnosed with early-stage cancer of one of eight common tumor types (ovary, liver, stomach, pancreas, esophagus, colorectal, lung, and breast) [80]. The median sensitivity of this test was 70% and the specificity was >99% [80]. A modified version of CancerSEEK was later employed in 10,000 healthy women, where it diagnosed 26 of them with cancer and, after PET-CT verification, it led to a curative surgery on 9 of them [81]. As several studies have shown that ctDNA is not detected in many early-stage breast cancer patients, even when ultra-highly sensitive techniques are applied, it makes more sense that plasma mutation testing becomes part of a more wholistic, minimally invasive modality.

In this review, we considered only the studies that examined ctDNA mutations; however, other features of cfDNA may be used in early-stage cancer diagnosis. Recent work showed that cfDNA methylation patterns had a high sensitivity at 98% specificity in early cancer detection and was the best predictor for the origin of cancer when different ctDNA features were evaluated [82]. Several studies have also shown that analysis of the cfDNA fragmentation pattern may improve the sensitivity of ctDNA assays (reviewed in [25,27]); as cfDNA in cancer patients is more fragmented than that of healthy individuals, a size selection step could enrich the sample in ctDNA. Furthermore, combination of the cfDNA fragmentation pattern and ctDNA mutations in machine learning algorithms could efficiently distinguish cancer and healthy populations, suggesting that it could be used for improved cancer detection (reviewed in [25,27]).

A reasonable question stemming from the above studies concerns the choice of the appropriate ctDNA analysis technique. There is a wide spectrum of platforms available ranging from different types of digital PCR to broader NGS-based panels. Even though the most suitable approach depends on the clinical question at hand, in our opinion, the power of sequencing to monitor simultaneously a multitude of tumor-specific variants and thereby reduce the risk of false-negative results offers a clear advantage over dPCR. Most of the studies described herein have employed patient-customized assays based on tumor profiling at diagnosis. Even though this approach allows for MRD detection, it is not well suited for capturing novel mutations that might arise during tumor evolution and under the pressure of therapy. For this purpose, a larger repertoire of genes needs to be interrogated in plasma and these results may inform efficacious therapeutic interventions.

The clinical trials underway are expected to provide the evidence and fine-tune the parameters to integrate ctDNA assays in decision-making in early-stage breast cancer.

## Figures and Tables

**Table 1:** Studies that performed ctDNA analysis for prediction of metastatic recurrence in early-stage breast cancer.

ctDNA Assay	No. of Patients ^#^	No. of Samples Analyzed	BrCa Subtype	Therapy Given	Lead Time before Clinical Relapse	Ref.
ddPCR (4–6 chromosomal rearrangements/patient)	20	93	mixed	AT	11 months	[65]
ddPCR (1–2 mutations/patient)	55	n/s	mixed	NAT	7.9 months	[66]
ddPCR (≥1 mutations/patient)	101	695	mixed	AT and/or NAT	10.7 months	[67]
Personalized NGS (16 mutations/patient)	49	208	mixed	AT and/or NAT or none	8.9 months	[68]
Personalized NGS (12–16 mutations/patient)	84	291	mixed	NAT	n/s	[59]
Personalized NGS (36 mutations/patient) *	83	219	HR^+^	AT	12.4 months	[69]
NGS (53 mutations/patient) *	142	271	mixed	AT and/or NAT	18.9 months	[70]
NGS (TARDIS) (18 mutations/patient) *	33	80	mixed	NAT and/or AT	n/s	[71]
NGS (Ion Ampliseq Oncomine Research Panel)	33	n/s	TNBC	NAT and AT	0.07–8.87 months	[72]
NGS (Foundation Medicine panels)	142	142	TNBC	NAT	n/s	[73]
dPCR (1–2 mutations/patient)	161	n/s	TNBC	NAT and AT	1.6 months (intervention group) 4.1 (observation group)	[74]

AT: adjuvant therapy; NAT: neoadjuvant therapy; n/s: not specified; HR: hormone receptor; * median number is shown; ^#^: number of patients whose plasma samples were analyzed.

**Table 2:** Current active clinical trials for ctDNA in early-stage breast cancer *.

Title of Study	Condition	Study Objective	Study Type/Design	Estimated Enrollment
HARMONY (NCT05433753)	HER2^+^ receiving neoadjuvant therapy	MRD detection using ctDNA to predict recurrence	observational, prospective	60
NCT04353557	stage I–III	assess the prognostic and predictive value of ctDNA	observational, prospective	200
NCT05649475	stage I–III undergoing NAT	whether ctDNA is a marker of response to NAT	observational, prospective	100
SURVIVE (NCT05658172)	medium- and high-risk patients after completion of primary anti-tumor therapy	evaluate the potential benefits of intensified versus standard surveillance	partially double-blinded, interventional, randomized, controlled, superiority study	3500
CIPHER (NCT05333874)	stage II–III TNBC and HER2^+^	examine the impact of ctDNA on treatment decision-making in patients after NAT and surgery	interventional, non-randomized	34 **
TRAK-ER (NCT04985266)	ER^+^	demonstrate that palbociclib and fulvestrant can defer or prevent relapse in patients with ctDNA-detected molecular relapse	interventional, randomized, phase II study	1100
DARE (NCT04567420)	stage II–III HR^+^/HER2^−^	assess the incidence of ctDNA detection in patients who receive standard-of-care endocrine AT and have high risk of recurrence and assess whether palbociclib plus fulvestrant improves relapse-free survival in ctDNA+ patients	interventional, randomized	100
LEADER (NCT03285412)	ER^+^	ctDNA for MRD detection and therapy guidance	interventional, randomized	120
NCT04768426	TNBC	evaluate the use ctDNA to identify patients who will or will not benefit from treatment with capecitabine	interventional, phase II study	25
NCT03881384	not specified	whether ctDNA detection can reflect the tumor response to NCT and detect MRD after surgery	observational, prospective	200
STRIVE (NCT03085888)	women undergoing mammography screening	validate the ability of the GRAIL Test to detect early-stage breast and other invasive cancers	observational, prospective	99,481 **
RENOVATE (NCT04781062)	women with radiologically identified lesions, BIRADS-3/4/5, smaller than 2 cm	develop a Horizontal Data Integration classifier enabling early noninvasive diagnosis	interventional, non-randomized	367 **
APOLLO (NCT04501523)	stage II-III TNBC	use ctDNA to identify patients with high relapse risk and randomize them to receive boost or standard therapy	interventional randomized, phase II	460
ARTEMIS (NCT04803539)	stage II-III TNBC	identify high-risk patients and initiate boost therapy	interventional randomized, phase II/III	120

* The terms “early breast cancer” or “stage I-III breast cancer” and “ctDNA” were used at clinicaltrials.gov and studies with the status “recruiting” and “active, not recruiting” are shown (1 May 2023). Manual inspection was performed to exclude inappropriate Studies. (** actual enrollment).

## Data Availability

No new data were created or analyzed in this study. Data sharing is not applicable to this article.
